# Evaluation of the Metered Dose Inhaler Technique: Initial Assessment and Post-counseling Improvements Among the Indian Population

**DOI:** 10.7759/cureus.57397

**Published:** 2024-04-01

**Authors:** Siddharth A, Raymond Haward, Ananya Chakraborty

**Affiliations:** 1 Endocrinology, Vydehi Institute of Medical Sciences and Research Centre, Bangalore, IND; 2 Internal Medicine, Vydehi Institute of Medical Sciences and Research Centre, Bangalore, IND; 3 Department of Pharmacology, Vydehi Institute of Medical Sciences and Research Centre, Bangalore, IND

**Keywords:** chronic obstructive pulmonary disease (copd), metered-dose inhaler (mdi), corrective education, post-intervention, pre-intervention, intervention, inhalation technique, asthma

## Abstract

Objective

The objective of this study was to evaluate errors in the use of metered-dose inhalers (MDIs) among patients diagnosed with asthma or chronic obstructive pulmonary disease (COPD). Additionally, we aimed to assess improvements following corrective interventions.

Settings and design

This cross-sectional study was done by simple random sampling.

Methods and materials

This study was done at a tertiary care center in South India in an outpatient department and ward for tuberculosis and chest disease to find out the right way to use an MDI and investigate the reasons why people with asthma and COPD don't use it correctly. There were a total of 12 steps. The patient was given an empty canister to try the inhalation technique and was scored one point for every correct step and zero for every incorrect step, for a total of 12 steps. Following the demonstration, an educator used a variety of tools, including verbal communication, pictorial demonstrations, and practical demonstrations, to correct the mistakes. After education was provided, post-interventional data was collected.

Results

During pre-intervention of the 12 steps out of the 183 participants, step one had 183 correct participants (100%), step two had 104 correct participants (56.83%), step three had 129 correct participants (70.49%), step four had 71 correct participants (38.79%), step five had 167 correct participants (91.25%), step six had 123 correct participants (67.21%), step seven had 132 correct participants (72.13%), step eight had 81 correct participants (43.71%), step nine had 123 correct participants (67.21%), step 10 had 108 correct participants (59.01%), step 11 had 128 correct participants (69.94%), and step 12 had 175 correct participants (95.62%). During the post-intervention of the 12 steps, out of the 183 participants, step one remained at 183 correct participants (100%), step two increased to 149 correct participants (81.42%), step three to step seven increased to 183 correct participants (100%), step eight increased to 142 correct participants (77.59%), step nine increased to 174 correct participants (95.08%), step 10 increased to 177 correct participants (96.72%), step 11 increased to 143 correct participants (78.14%) and step 12 increased to 177 correct participants (96.72%).

Conclusion

This study highlights the prevalent errors in the use of metered-dose inhalers (MDIs) among patients diagnosed with asthma or chronic obstructive pulmonary disease (COPD). The results demonstrate significant improvements in the MDI technique. Following educational interventions such as verbal communication, pictorial demonstrations, and practical exercises, patients were able to correct their inhaler technique effectively and emphasized the importance of patient education and counseling to ensure the maintenance of correct usage over time.

## Introduction

In today's world, asthma and chronic obstructive pulmonary disease (COPD) are two of the most common diseases we come across in respiratory system pathology. These diseases are chronic in nature, and medications remain the mainstay of treatment. Patients are prescribed anticholinergics like ipratropium bromide and triotropium bromide, beta-2 adrenergic agonists like salbutamol, salmetrol, terbutaline, and formoterol, and inhalation steroids like fluticasone and budesonide to improve their symptoms [1]. These medications are administered through metered-dose inhalers (MDIs). There are a lot of advantages to the use of MDI, such as financial affordability, convenience, portability, quickness, local action, and negligible systemic side effects [2].

The method of administering the inhalation drug is crucial in order to get the optimum delivery and the desired effect. The efficacy of the MDI decreases, and the symptoms aggravate due to the inappropriate technique [3]. Lack of knowledge is attributed to the cause [4]. Education is the key to enhancing the administration techniques.

Many studies have been done to evaluate the use of drugs in the treatment of asthma and COPD. Only a few studies persist in evaluating the technique of using MDI [5]. This study was done to look at the steps missed by patients to effectively deliver the MDI. Appropriate education intervention was provided to the patients who performed the wrong technique of using MDI after initially observing the steps. After the education intervention was provided, the patient was reassessed to assess the improvement in the technique of using MDI.

## Materials and methods

This cross-sectional study was conducted at the outpatient department and the ward of tuberculosis and chest disease in a tertiary hospital in South India, Vydehi Hospital, for patients who had asthma or COPD. The study duration was three months. Prior permission was obtained from the Institutional Ethics and Research Committee of Vydehi Institute of Medical Sciences and Research Centre, Bangalore. Anonymity and confidentiality were maintained by not including the names of the participants, and the data was not accessible to anybody except the researchers and the statistical support unit. Written, informed consent was obtained from the participants. Participants had the right to refuse to participate at any point in the study. The demographic details, such as name, age, gender, smoking habits, educational status, monthly income, duration of disease, details, and duration of drug treatment, were recorded. The patient's knowledge about the inhaler technique and the method of demonstration was also recorded. The patient was provided with a placebo MDI device with an empty canister and was asked to demonstrate the inhalation technique. No oral instructions, prompts, or critiques were provided prior to, during, or after the demonstration. The technique was scored as per a checklist based on the 12 steps of use of MDI recommended by the WHO [6]. 

The patients' inhaler usage practices are examined using a set of 12 steps, as shown in Table 1.

**Table 1:** Recommended 12 steps for MDI utilization by WHO Checklist by the World Health Organization [6] MDI - metered-dose inhalers

Step number	Step description
1	Remove the cap.
2	Check the dose counter.
3	Hold the inhaler upright and shake it well.
4	Breathe out gently through the mouth (away from the inhaler).
5	Put the mouthpiece of the inhaler between the teeth and close the lips to form a good seal.
6	Start to breathe slowly through the mouth, and at the same time, press down firmly on the canister.
7	Continue to breathe in slowly and deeply.
8	Hold the breath for 10 seconds or as long as comfortable.
9	While holding the breath, remove the inhaler from the mouth.
10	Breathe out gently through the nose (away from the inhaler).
11	If more doses are needed, repeat steps 2–9.
12	Replace the cap.

A score of one was assigned to each step performed correctly, whereas a wrongly performed step was allotted a score of zero. After patients demonstrated their technique, their mistakes were pointed out, and education was provided for the proper technique of use of the meter-dose inhaler using various tools such as verbal communication, pictorial demonstrations, and practical demonstrations. After education was provided, the patient was reassessed immediately, and a score was given post-intervention.

Inclusion criteria

Adult patients >18 years who were diagnosed with suffering from asthma or COPD, were currently using a manually operated MDI, and were ready to provide informed consent were included in this study.

Exclusion criteria

Pregnant women and children, patients with respiratory diseases other than asthma or COPD, those who were not prescribed drugs that required the use of MDI, and those who did not self-administer their MDI were excluded from the study.

Statistical analysis

The data was coded and entered on an Excel sheet (Microsoft, Redmond, Washington). Statistical analysis was performed and analyzed using Statistical Package for Social Sciences (SPSS) version 23 (IBM Inc., Armonk, New York). The mean and standard deviation, or median, were used to present continuous variables, while frequency and percentage were used to present categorical variables. The descriptions of the data were in tables and graphs. The Chi-squared test, or Fisher's exact test, was done for associations. A p-value of <0.05 was considered significant.

Sample size

The sample size was calculated by using the expected proportion of 0.65, precision of 7%, confidence interval of 95%, and the required minimum sample size of 178. The sample strategy was the line listing of all the patients diagnosed with asthma or COPD in Vydehi Hospital, Whitefield area. By simple random sampling, every Nth person who met the inclusion criteria was enrolled in the study.

## Results

The patients details before testing the 12 steps were collected based on demography, symptomology, and examination (Table 2).

**Table 2:** Demography, patient symptoms, and examination findings of study participants PUC - pre-university course; SSLC - secondary school leaving certificate; COPD - chronic obstructive pulmonary disease; MDI - metered-dose inhalers

Questions		Distribution of participants based on their characteristics	Total frequency (n)
Age in years (range)	18-30	30 (16.4%)	n = 183
	31-40	28 (15.3%)	
	41-50	31 (16.9%)	
	51-60	26 (14.2%)	
	61-70	29 (15.8%)	
	71-80	39 (21.3%)	
Gender	Male	118 (64.4%)	
	Female	65 (35.6%)	
Education	Professional	36 (19.7%)	
	Graduate	23 (12.6%)	
	PUC	10 (5.5%)	
	SSLC	5 (2.7%)	
	Middle school	34 (18.6%)	
	Primary school	64 (35%)	
	Illiterate	11 (6%)	
Occupation	IT	11 (6%)	
	Business	52 (28.4%)	
	Accountant	10 (5.5%)	
	Clerk	9 (4.9%)	
	Farmer	33 (18%)	
	Homemaker	43 (23.5%)	
	Maid	6 (3.3%)	
	Security	5 (2.7%)	
	Social worker	12 (6.6%)	
	Peon	2 (1.1%)	
Marital status	Married	170 (92.9%)	
	Unmarried	13 (7.1%)	
Socioeconomic status	Upper class	7 (3.8%)	
	Upper-middle class	44 (24%)	
	Upper-lower class	26 (14.2%)	
	Lower-middle class	93 (50.8%)	
	Lower class	13 (7.1%)	
Chief complaints	Breathlessness	2 (1.1%)	
	Chest tightness	12 (6.6%)	
	Cough, breathlessness, expectoration	18 (9.8%)	
	Cough, breathlessness, hemoptysis	11 (6%)	
	Cough, breathlessness	83 (45.4%)	
	Cough, wheezing, chest tightness	43 (23.5%)	
	Cough, fever, weight loss	1 (0.5%)	
	Breathlessness, chest tightness	4 (2.2%)	
	Hemoptysis, cough	3 (1.6%)	
	Wheezing	6 (3.3%)	
Past history	Nil	120 (65.6%)	
	Tuberculosis, hypertension	1 (0.5%)	
	Tuberculosis	5 (2.7%)	
	Hypertension	23 (12.6%)	
	Diabetes, hypertension	10 (5.5%)	
	Diabetes	24 (13.1%)	
Smoking history expressed in 10-year intervals	Nil	68 (37.2%)	
	1-10	26 (14.2%)	
	11-20	29 (15.8%)	
	21-30	32 (17.5%)	
	31-40	18 (9.8%)	
	41-50	10 (5.5%)	
Clubbing	Present	23 (12.6%)	
	Absent	160 (87.4%)	
How many years have passed since the initial diagnosis of asthma or COPD?	1-10	97 (53%)	
	11-20	57 (31.1%)	
	21-30	20 (10.9%)	
	31-40	7 (3.8%)	
	41-50	2 (1.1%)	
Frequency of symptoms	Seasonal	131 (71.6%)	
	Non-seasonal	52 (28.4%)	
Inhaler composition	Formetrol fumarate and budesonide	42 (23%)	
	Levosabutamol and ipratropium	32 (17.5%)	
	Tiotropium and formetrol	12 (6.6%)	
	Salbutamol and betamethasone	15 (8.2%)	
	Salmetrol and fluticasone	71 (38.8%)	
	Salbutamol	11 (6%)	
Duration of inhaler use in years (range)	1-10	113(61.7%)	
	11-20	54 (29.5%)	
	21-30	12 (6.6%)	
	31-40	3 (1.6 %)	
	41-50	1 (0.5%)	
Frequency of change in inhaler composition	No	64 (35.0%)	
	Once	9 (4.9%)	
	Twice or more	110 (60.1%)	
Inhaler was explained by whom?	Doctor	64 (35.0%)	
	Nurse	9 (4.9%)	
	None	110 (60.1%)	
Was inhaler use demonstrated to the patient?	Yes	49 (67.1%)	n = 73
	No	24 (32.9%)	
Did the patient demonstrate back the use of inhaler?	Yes	18 (36.7%)	n = 49
	No	31 (63.3%)	
The form in which education was given	Verbally	68 (93.2%)	n = 73
	Chart	5 (6.8%)	
Who monitors the use of inhaler?	Daughter	26 (14.2%)	n = 183
	Son	35 (19.1%)	
	Father	4 (2.2%)	
	Mother	9 (4.9%)	
	Sister	2 (1.1%)	
	Husband	42 (23%)	
	Wife	65 (35.5%)	
Was the patient confident to use the inhaler?	Confident	174 (95.1%)	
	Not confident	9 (4.9%)	
Does the patient know when the MDI is empty	Yes	178 (97.3%)	
	No	5 (2.7%)	

**Figure 1:**
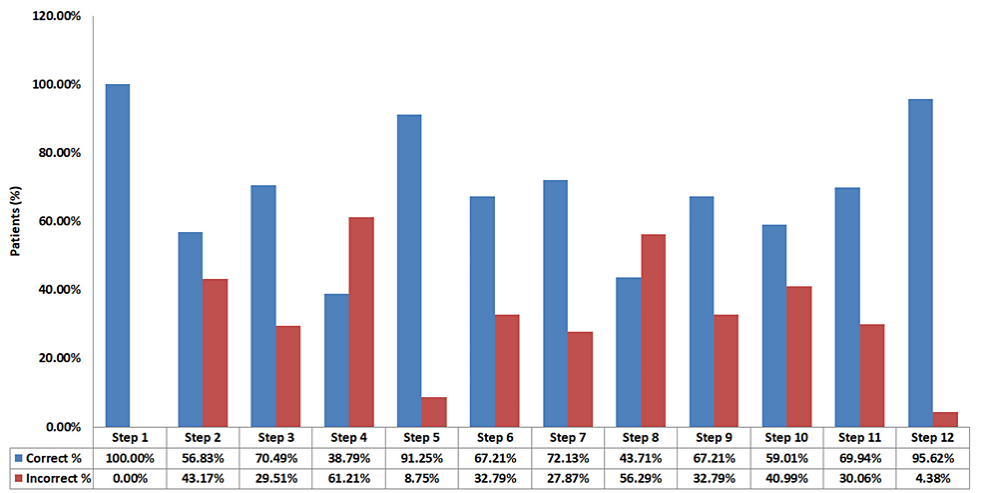
Evaluation of patients' MDI steps before intervention MDI - metered-dose inhalers

During pre-intervention of the 12 steps out of the 183 participants, step one had 183 correct participants (100%), step two had 104 correct participants (56.83%), step three had 129 correct participants (70.49%), step four had 71 correct participants (38.79%), step five had 167 correct participants (91.25%), step six had 123 correct participants (67.21%), step seven had 132 correct participants (72.13%), step eight had 81 correct participants (43.71%), step nine had 123 correct participants (67.21%), step 10 had 108 correct participants (59.01%), step 11 had 128 correct participants (69.94%), and step 12 had 175 correct participants (95.62%; Figure 1).

**Figure 2:**
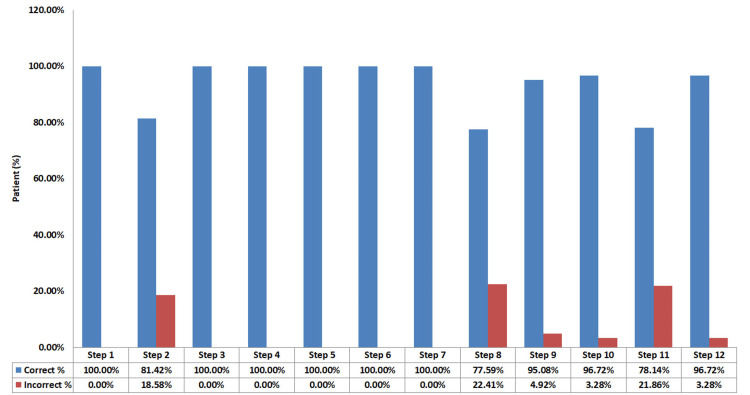
Evaluation of patients' MDI steps after intervention MDI - metered-dose inhalers

During the post-intervention of the 12 steps, out of the 183 participants, step one remained at 183 correct participants (100%), step two had increased to 149 correct participants (81.42%), step three to step seven increased to 183 correct participants (100%), step eight increased to 142 correct participants (77.59%), step nine increased to 174 correct participants (95.08%), step 10 increased to 177 correct participants (96.72%), step 11 increased to 143 correct participants (78.14%), and step 12 increased to 177 correct participants (96.72%; Figure 2).

## Discussion

At present, inhalers are a widely used medication for asthma and COPD patients. It provides quick relief by delivering medication directly to the lungs [7]. Inhalers are portable, easy to use, and offer a convenient way for individuals to manage their respiratory conditions. Additionally, inhalers have improved over the years with the development of new technologies, such as digital trackers and sensors, which can help patients keep track of their medication usage and monitor their symptoms more effectively [8]. As a result, inhalers have become an essential tool in the management and treatment of asthma and COPD.

Studies show that despite using an inhaler, some patients do not show improvement in the symptoms due to poor drug delivery to the bronchial smooth muscles. This is because many patients do not use their inhalers correctly, leading to improper drug delivery [9]. This was noticed in our study while evaluating the 12 steps on the technique of drug delivery in MDI.

All of the patients were able to complete the first step, which was removing the cap from the inhaler. In the second step, the evaluation of the dose counter revealed a notable improvement after emphasizing its significance. A minority of patients failed to adhere to this procedure (p-value=0.344), possibly due to their awareness of having already completed it either during the preintervention stage or when the healthcare worker identified the mistake during the remedial phase.

During steps three to seven of the intervention, no errors were detected, indicating that the education provided effectively contributed to the improvement of error rates. The p-values cannot be reported as the post-intervention all the participants did these steps without errors. In the eighth step, a significant improvement in the recollection of the technique was noted (p<.001). A few patients were not able to hold their breath for more than 10 seconds. It may be due to reduced lung function due to asthma or COPD. Older patients may have difficulty coordinating the steps in sequence due to impaired motor skills [10]. The ninth step, which involved the voluntary action of holding one's breath for a duration of five seconds or till reaching a comfortable limit, exhibited few errors in the post-intervention phase but with remarkable improvement (p<.001). These difficulties may be attributed to the presence of severe pathological conditions in specific patients, hence impeding their ability to sustain breath-holding [11]. Step 10 (p=0.003), which involved the removal of the inhaler from the mouth and the gentle exhalation away from the inhaler via the nose, showed significant improvement. Step 11, which involves repeating the process from step three to step 10 after finishing step 10, revealed the presence of faults that persistently recur unless additional corrective measures are taken. The faults were subsequently clarified to the participants, taking into account the errors that remained present following the initial demonstration of their original errors (p<.001). The correction of step 12, which involves replacing the cap, is of significance (p<.001) because of its potential simplicity in recall. This step serves the purpose of safeguarding against contamination, preserving the drug's quality, and facilitating the distinction of the inhaler from others.

Healthcare professionals play a vital role in educating patients on the proper use of inhalers, especially about mistakes and confusion regarding the technique [12]. There was a significant improvement in the inhaler technique after correcting the subject's errors. By ensuring patients understand how to use their inhalers correctly, healthcare providers can help improve drug delivery and ultimately enhance symptom management for asthma and COPD. Lack of clear instruction by healthcare workers can be a cause for mistakes and confusion regarding MDI usage [13]. Also, cleaning and replacing the clogs or expired devices must also be advised to the patients, as these factors also lead to poor outcomes in controlling their pathology [14].

Practical demonstration and "teach-back" methods are the cornerstones of improving the technique [15]. Practical demonstrations on how to use the inhaler properly can help patients understand the correct steps and reduce the risk of errors [16]. Additionally, receiving feedback from healthcare professionals on their technique allows patients to address any misunderstandings or incorrect practices. Implementing these strategies can empower patients to take control of their medication management and improve their overall treatment outcomes. 

The teach-back method can further enhance patient comprehension by encouraging them to repeat the instructions back to the healthcare provider [17]. This method ensures that patients fully understand the medication instructions and can effectively follow them. It also allows healthcare providers to identify any areas of confusion or misunderstanding and address them promptly [15]. By actively involving patients in their own healthcare, the teach-back method promotes better medication adherence and reduces the risk of medication errors. Also, giving printed materials or handouts showing adequate steps can help the patient recall their mistakes significantly [15].

Patients were also observed to have poor coordination while inhaling the medication, leading to suboptimal drug delivery [18]. One potential solution for patients with poor coordination while inhaling medication is the use of spacer devices, which can help improve drug delivery and ensure that the medication reaches the intended target in the lungs [18]. Another strategy to address poor coordination during inhalation is to provide thorough demonstrations and instructions on proper inhaler technique [19]. Healthcare professionals can do this during clinic visits or by providing patients with access to instructional videos at home [19]. It may also be beneficial to conduct regular check-ins with patients to assess their progress.

The education of the patient must be taken into consideration while explaining the steps [20]. The healthcare provider should ensure that the patient understands the importance of proper inhaler technique and the potential consequences of not using the inhaler correctly [18]. They should also address any questions or concerns the patient may have and provide additional resources or support if needed [18]. Regular check-ins with patients can help identify any difficulties or challenges they may be facing in using their inhalers correctly, allowing for timely intervention and adjustment of the treatment plan if necessary.

Although spacers can enhance optimal delivery, they are recommended for all patients using inhalers. However, patients don't use spacers due to multifactorial causes [21]. It may be due to healthcare workers not prescribing one for them or subjects believing that MDI alone is more effective [22]. Besides, a spacer is difficult to carry when compared to an inhaler, which is compact and easy to carry in a bag or a pocket [23]. This leads to the development of the habit of using MDI without a spacer. 

The strength of the study highlights the errors found in the use of MDI and the improvement post-correction of patients, which highlights the importance of effectively delivering the information through demonstrations and the teach-back method and the study encompassed participation from both genders in its findings.

The limitation of this study was that no follow-up was done with regard to the patients who used the inhaler incorrectly to assess long-term retention of the instructions for adequate drug compliance. This study was a single-center study. It is also a cross-sectional study showing a snapshot scenario of the patients following up on the steps. The study was conducted only on patients who visited hospitals seeking medical care.

## Conclusions

This study highlights the prevalent errors in the use of metered-dose inhalers (MDIs) among patients diagnosed with asthma or chronic obstructive pulmonary disease (COPD). The results demonstrate significant improvements in the MDI technique. Following educational interventions such as verbal communication, pictorial demonstrations, and practical exercises, patients were able to correct their inhaler technique effectively. These findings emphasize the importance of comprehensive patient education and counseling in optimizing the management of asthma and COPD. Healthcare providers should prioritize regular assessment of inhaler technique and provide ongoing support to ensure patients maintain correct usage over time.
